# A Newly Established ELISA for the Surveillance of Rift Valley Fever in Dromedary Camels and Their Owners, Kenya 2018

**DOI:** 10.3390/v18040445

**Published:** 2026-04-08

**Authors:** Shannon L. M. Whitmer, Jessica Rowland, Emir Talundzic, Deborah Cannon, Aridth Gibbons, Cynthia Ombok, Jennifer L. Harcourt, Natalie J. Thornburg, Clayton Onyango, Peninah Munyua, Elizabeth Hunsperger, Isaac Ngere, M. Kariuki Njenga, Caroline Ochieng, Mathew Muturi, Joel M. Montgomery, Marc-Alain Widdowson, John D. Klena

**Affiliations:** 1 Viral Special Pathogens Branch, Centers for Disease Control and Prevention, 1600 Clifton Rd. NE, Atlanta, GA 30329, USAztq9@cdc.gov (J.M.M.); irc4@cdc.gov (J.D.K.); 2 Washington State University Global Health Program, Washington State University, One Padmore Place, Off Ngong Road, Nairobi 00200, Kenya; 3 Coronavirus and Other Respiratory Viruses Laboratory Branch, Centers for Disease Control and Prevention, 1600 Clifton Rd. NE, Atlanta, GA 30329, USA; 4 Centers for Disease Control and Prevention—Kenya, KEMRI Complex, Mbagathi Road Off Mbagathi Way, Village Market, Nairobi P.O. Box 606-00621, Kenya; 5 Center for Global Health Research, Kenya Medical Research Institute, Kisumu P.O. Box 1578-4100, Kenya; 6 Zoonotic Disease Unit, Kenya Ministry of Agriculture, Livestock, Fisheries and Cooperatives, Nairobi P.O. Box 30028-00100, Kenya; 7 Department of Veterinary Medicine, Dahlem Research School of Biomedical Sciences (DRS), Freie Universität Berlin, 14195 Berlin, Germany

**Keywords:** Rift Valley fever virus, seropositivity, IgG, humans, camels, infectious disease, zoonotic spread

## Abstract

In 2024 Kenya had a population of 4.78 million camels that contributed to the livelihoods of pastoralist communities in northern Kenya. Previous studies in Kenya, Saudi Arabia and eastern Africa demonstrated high seroprevalence of Middle East respiratory syndrome coronavirus (MERS-CoV)-specific antibodies in dromedary camels, as well as sporadic transmission of MERS-CoV from camels to humans. Based on the MERS-CoV data and the very close contact between owners and their camels in northern Kenya, we speculated that camels may also transmit other zoonotic viruses, such as Rift Valley fever virus (RVFV). In this study, 493 camel and 197 human sera were collected in Marsabit, Kenya, through a cross-sectional survey in 2018 and analyzed for the presence of RVFV IgG antibodies using a laboratory-developed indirect enzyme-linked immunosorbent assay (ELISA). Overall, 15.6% of camels and 7.6% of humans were RVFV IgG-positive; IgG-positive camels were predominantly females in large population herds and IgG-positive humans were engaged in farming-related activities and were greater than 18 years old. Of the eight location groups sampled, two had high camel (site 2 and site 6) and two had high human (site 5 and site 6) RVFV seropositivity rates. These data suggest that camelids, such as dromedary camels, may serve as amplifying hosts for vector-borne zoonotic diseases, such as RVFV, and that humans with frequent farming and camel meat, milk, or camel product contact may have increased risk for RVFV exposure or infection.

## 1. Introduction

In Kenya, rising temperatures and unpredictable droughts have forced more pastoralists and farmers from cattle to camel herding [1]. This shift has now established Kenya as the second highest producer of camel milk worldwide and serves as the primary source of income for many farmers [2]. In 2024, Kenya had a population of 4.78 million camels that contributed to livelihoods of pastoralist communities in northern Kenya [3]. The weak enforcement of existing health and safety guidelines for camel products creates a potential public health risk to consumers; this risk includes exposure to camel-borne zoonotic diseases; the economic impact of a camel-related outbreak could substantially affect the camel product value chain in Kenya [2].

Northern Kenya has been the primary location for farming dromedary camels but camel farming has recently expanded to central and southern Kenya [4]. A proportion of farmed camels are exported from Kenya to Saudi Arabia, United Arab Emirates (UAE), Qatar, and Yemen to be used for meat, milk, cultural, or recreational (racing, parades, or festivals) purposes [5]. Studies of the camel-borne Middle East respiratory syndrome coronavirus (MERS-CoV) have demonstrated the potential for dromedary camels to act as a zoonotic reservoir for human infection [6,7,8]. In Saudi Arabia, camel farmers and slaughterhouse workers who came in contact with body fluids from camels (milk, meat, nasal and oral secretions) were more likely to become seropositive for MERS-CoV-specific antibodies [9,10,11].

While much is known about dromedary camel-mediated MERS-CoV spread and transmission, little is known about Rift Valley fever virus (RVFV) seroprevalence in Kenyan camels and the seroprevalence of the camel owners. RVFV transmission to humans often occurs through the handling or consuming of meat, blood and other body fluids from an infected animal, or through mosquito vectors, but it is currently unknown as to whether camel owners are also at risk for RVFV infection [12,13]. Similar to MERS-CoV, Kenyan farmers that milked or consumed raw milk from cows, sheep, or goats had an increased risk of previous infection with RVFV [14]. RVFV is endemic in many countries in Africa (more than 30 countries) and the Middle East [15]. In humans, RVFV infections mimic influenza-like symptoms such as fever, headache, abdominal pain, nausea and vomiting [16,17]. However, in a small percentage of humans (estimate ≤5%), infection can progress to a much more severe form of disease with hemorrhagic manifestations, ocular complications, meningoencephalitis, and death [18].

In this study, a camel-specific RVFV IgG ELISA was developed and serological evidence gathered to understand the frequency of previous infections with RVFV (or antigenically similar Phleboviruses) in Kenyan dromedary camels. Human camel owners were also evaluated for the presence of RVFV-specific IgG using a human-specific RVFV ELISA. Altogether, these data demonstrate that camels can be infected with RVFV and could serve as an intermediate species capable of amplifying and spreading the vector-borne disease.

## 2. Materials and Methods

### 2.1. Specimen Collection and Sampling Groups

Human and camel specimens were collected as part of a serosurvey evaluating the prevalence of MERS-CoV antibodies [8]. After completion, MERS-CoV testing residual specimens were shared with the Viral Special Pathogens Branch (VSPB) in Atlanta, Georgia, USA, for evaluation of RVFV seroprevalence. 

### 2.2. RVFV Indirect ELISA to Detect Camel IgG-Specific Antibodies

Prior to performing the ELISA, camel serum specimens were gamma irradiated with 5 × 10^6^ rads, heat inactivated at 56 °C for 30 min and incubated for 30 min at 37 °C in cow milk master plate diluent (MPD) (5% cow milk powder, 1× phosphate-buffered saline (PBS) with 0.5% Tween-20). RVFV-negative bovine sera and sera from North American dromedary camels (RVFV naive, kindly provided by Richard Bowen at Colorado State University) were included as negative controls. RVFV-positive bovine serum from the Uganda Virus Research Institute (UVRI) was used as a positive ELISA control because an RVFV-positive dromedary serum specimen was not available. Individual ELISA plate wells were coated with whole-cell RVFV antigens (RVFV-infected Vero E6 cell lysate in 1% triton X-100 in borate saline, pH 9.0) [19,20] or uninfected Vero E6 cell lysate at 1/2000 dilutions and incubated at 4 °C for 24 h. Plates were washed three times with 1× PBS containing 0.1% Tween-20 and 100 µL goat milk serum diluent (1× PBS, 5% goat skim milk powder, and 0.1% Tween-20) was added. Sera in MPD was added (33 µL) and titrated following a 4-fold dilution series (1:100, 1:400, 1:1600, 1:6400). Primary antibodies were adsorbed for 1 h at 37 °C. After incubation, plates were washed 3 times with 1× PBS and protein A/G-HRP (1:1500, Thermofisher, Waltham, MA, USA), anti-Llama HRP (1:8000 dilution, Agrisera, Vännäs, Sweden, AS101420, cross-reacts with camelid species [21,22]) or anti-bovine HRP (1:1000 dilution, KPL, Milford, MA, USA, 14-12-06) was added to serum diluent and 100 µL was added to plates. Secondary antibodies were absorbed for 1 h at 37 °C, plates were washed three times with 1× PBS, and 100 µL of ABTS Peroxidase substrate (KPL, Milford, MA) was added and plates were incubated for 30 min at 37 °C. Optical density (OD) absorbance at 414 nm (specific signal) and 490 nm (background plate signal) was read using a plate reader (BioTek PowerWave 340, Agilent, Santa Clara, CA, USA).

### 2.3. Determination of Camel RVFV IgG-Positive and -Negative Criteria

Two selection criteria were established for a camel specimen to be classified as RVFV IgG-positive: (1) the camel signal intensity (i.e., sum and adjusted OD values) must be greater than 3 standard deviations above background and camel negative control OD values; (2) dose-dependent antibody titration occurred over the dilution series. Adjusted OD values were calculated by subtracting the Vero E6 lysate OD values (background OD) from the RVFV Vero E6 lysate OD values (sum OD) for all serial dilutions tested. Adjusted sum OD values were calculated by totaling the adjusted OD values for the four serial dilutions (1/100, 1/400, 1/1600, 1/6400). To determine evidence of dose-dependent titration, adjusted OD values and dilution ratio were log10 transformed and the linear trendline slope and Pearson’s correlation coefficient (Pearson’s ρ) were calculated in Excel from the 1:100 dilution to the highest dilution that met the signal intensity threshold. If the titration slope was less than −0.2 and the Pearson’s ρ value was between −0.5 and −1 (moderate to strong linear correlation), there was evidence of a dose-dependent antibody titration. Specimens that did not serially dilute (slope was >−0.2) and/or had a Pearson’s ρ value greater than −0.5 (indicating a poor linear relationship between dilutions) were reviewed on a case-by-case basis to evaluate whether antibody-dependent competition at the lowest dilution (1:100) reduced the OD. Specimens that did not meet these criteria were defined as negative. Equivocal samples were either (1) positive only at the first dilution and met the adjusted SUM OD threshold or (2) positive at the 1/100 and 1/400 dilutions but did not meet the adjusted SUM OD threshold.

### 2.4. RVFV Indirect ELISA to Detect IgG-Specific Antibodies in Human Sera

Human sera were inactivated for 30 min at 37 °C in MPD. Assays were performed as reported in a previous study [17].

### 2.5. RVFV Quantitative Real-Time PCR Assays

RNA was extracted from camel sera using a MagMax™ Pathogen RNA/DNA extraction kit (Applied Biosystems, Waltham, MA, USA). RNA was pooled 8-fold and the presence of RVFV RNA was targeted using a qRT-PCR assay. RVFV RNA was detected using RVFV-F-2912 (5′ TGA AAA TTC CTG AGA CAC ATG G 3′), RVFV-R-2981 (5′ ACT TCC TTG CAT CAT CTG ATG 3′), and RVFV-PROBE-2950 (5′/56-FAM/CAC AAG TCC/ZEN/ACA CAG GCC CCT TAC ATT G/3IABkFQ/3′) with the following cycling conditions for 40 total cycles: 50 °C for 30 min, 95 °C for 2 min, 95 °C for 15 s and 60 °C for 45 s. Total RNA quality was assessed using eukaryotic 18S rRNA endogenous control (4319413E, Thermofisher, Waltham, MA, USA) with the following cycling conditions for 40 total cycles: 50 °C for 15 min, 95 °C for 2 min, 95 °C for 15 s and 55 °C for 45 s. RNA extraction lysis buffer only and water were included as negative extraction and negative template controls in the RVFV and 18S rRNA reactions. RNA extracted from the sera of two camels reared in the US (RVFV-negative) were also included as RVFV-negative controls and 18S rRNA endogenous positive controls. RNA extracted from RVFV-infected Vero E6 cells (used to produce RVFV antigens) was included as an RVFV-positive template control.

### 2.6. Statistical Analysis

Univariate analysis of factors associated with RVFV IgG seropositivity were calculated using Jupyterhub (version 1.5.0) and R (version 4.1.3). The χ^2^ (continuity correction = FALSE) and Fisher exact test (including Odds Ratios) were calculated using 2 × 2 contingency tables of available factors. For herd association tests, location 4 was used as the reference location due to the initial assumption of low RVFV prevalence across northern Kenya.

### 2.7. Spatial Distribution of RVFV IgG-Seropositive Camels and Humans

Camels and humans were enrolled at different time points throughout the study; therefore, closeness of geocodes at enrollment, herds that grazed together, and similarity in household member details were used to link camel and humans to a locality number. RVFV IgG group seroprevalence was determined by using total number of seropositive individuals at a locality/total number of individuals in group at a locality. The study area and distribution of sampling groups for camels and humans were mapped using ArcGIS Pro 3.1.4 (Redlands, CA, USA) and land cover was projected using GlobCover2009 (ESA and the Université Catholique de Louvain).

## 3. Results

### 3.1. Development of RVFV-Specific Camel IgG ELISA

**Figure 1:**
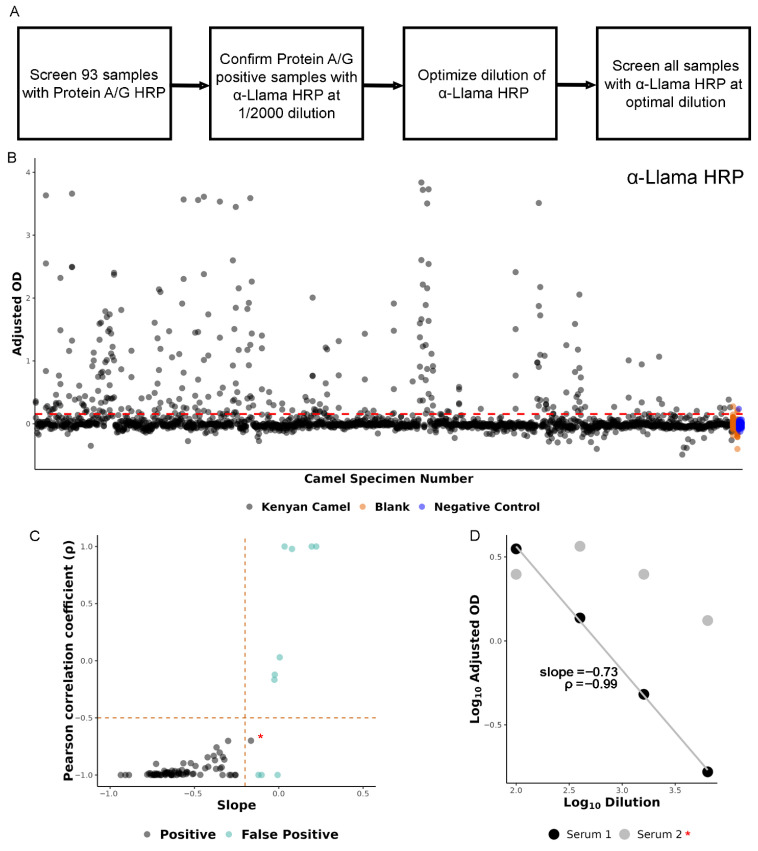
Development of RVFV-specific camel IgG ELISA. (**A**) Overview of methodology to develop RVFV-specific camel IgG ELISA. (**B**) Distribution of RVFV adjusted optical density (OD) values for serially diluted specimens. Dashed red line represents signal intensity threshold set at three standard deviations above background (orange points) and negative control camel (blue points) adjusted OD values. (**C**) Evaluation of titratable effect for specimens exceeding the signal intensity threshold set in (**B**) (dashed red line in (**B**)). Dashed orange lines indicate thresholds set for slope and Pearson’s ρ values. Point color indicates positive and false-positive results. All false-positive results were reviewed by hand to evaluate for evidence of competitive exclusion, seen in one sample (red star). (**D**) Example of dilution series versus adjusted OD values for two serum specimens. Slope and Pearson’s ρ are included in the figure for serum 1 (black points). Serum 2 is positive and exhibits evidence of competitive exclusion at the lowest dilution ratio, seen in (**C**) (red star).

To develop a camel IgG-specific RVFV ELISA, we adapted an approach previously used to detect human IgG and IgM antibodies targeting RVFV [17]. RVFV antigens were coated onto ELISA plates, however the detection method was modified from α-human IgG HRP to Protein A/G-HRP [23] or α-Llama IgG HRP. Initially, a subset (*n* = 93) of the 493 camel sera were screened with protein A/G-HRP to identify positive samples with an optical density (OD) greater than three standard deviations above background and naïve (US-born) camel absorbance values (Figure 1A and Figure S1A,B) [24,25,26]. Cross-reactivity (measured as the OD obtained using Vero E6 cells not infected with RVFV) was subtracted from the OD obtained from the RVFV-infected Vero E6 cells (Supplementary Figure S1A, “adjusted OD”); sera was serially diluted (1/100, 1/400, 1/1600, and 1/6400) and absorbance at all dilutions was assessed (Supplementary Figure S1B, “Sum OD”). This approach identified 7/93 positive and 3/93 nearly positive (“equivocal”) camel sera (Supplementary Figure S1A,B). To support these initial exploratory results, a subset (three strong Protein A/G anti-RVFV-positive specimens and US camel negative control specimens) of the protein A/G-positive camel sera were re-screened using α-Llama HRP which was optimized to identify the dilution with the lowest cross-reactivity to the Vero E6 cell lysate (1:8000 dilution) (Figure 1A).

After optimization of the α-Llama HRP secondary antibody, the entire camel sera sample set (*n* = 493) was screened for the presence of RVFV antibodies. A positive sample was defined as one exceeding the background and negative control signal intensity threshold by three standard deviations (red dashed line, Figure 1B and Figure S1C) and exhibiting a titratable response (Figure 1C,D). The titratable response was measured by fitting a linear trendline to all positive dilutions for each sample (Figure 1D) and plotting the trendline slope versus the Pearson covariance coefficient (a measure for how well the linear trendline fit the data) (Figure 1C). For example, Figure 1D demonstrates the relationship between the linear trendline slope and Pearson’s ρ values for a sample with a perfect dilution series (black) and a sample exhibiting competitive exclusion at the highest dilution (grey). Samples that crossed the signal intensity threshold, but exhibited a positive slope as dilution ratio increased and positive-to-weak covariance coefficient, were defined as false-positive (Figure 1C—green points).

### 3.2. RVFV Seroprevalence in Camels and Human Owners in Northern Kenya

**Figure 2:**
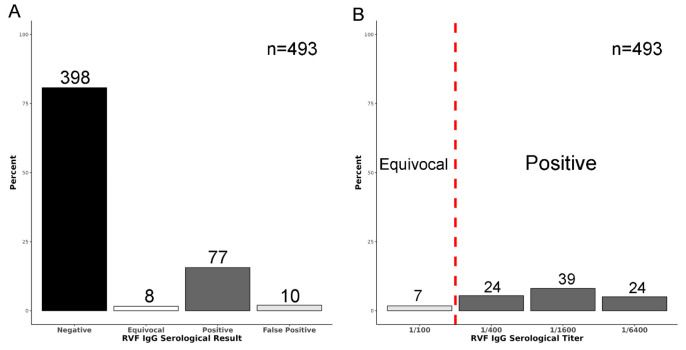
Summary of anti-RVF camel IgG serological testing. (**A**) Percentage and raw values (above bars) of negative, equivocal, positive, and false-positive results. Negative samples were below the signal intensity threshold values, while positive samples met the signal intensity thresholds and exhibited evidence of a titratable response. Equivocal samples either met the signal intensity threshold for the lowest dilution and SUM OD or for the lowest dilutions, but not the SUM OD. False-positive values met the signal intensity threshold, but did not exhibit a titratable response, seen in Figure 1C. (**B**) Summary of anti-RVFV camel IgG-serological-positive and -equivocal results, separated by titer.

After applying the signal intensity and titratable response criteria, 80.7% (398/493) of camel serum samples were considered negative and 15.6% (77/493) were considered positive (Figure 2A). One percent (1.6%, 8/457) of samples were classified as equivocal (Figure 2A,B). Equivocal samples were either (1) positive only at the first dilution and met the SUM OD threshold (1:100, *n* = 7) or (2) positive at the 1/100 and 1/400 dilutions but did not meet the SUM OD threshold (*n* = 1). Two percent (2.0%, 10/493) of samples were classified as false-positive since they met the signal intensity threshold values but did not exhibit evidence of titration (either due to a positive/flat slope between increasing dilutions, or due to high covariance between dilutions) (Figure 1C and Figure 2A).

**Figure 3:**
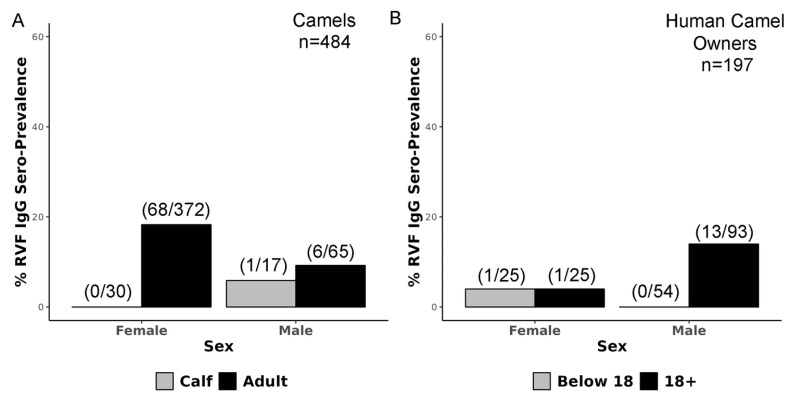
RVF seroprevalence in camels and human camel owners in northern Kenya. (**A**) Camel RVFV IgG seroprevalence stratified by age (calf ≤12 months, adult >12 months) and sex. Numbers above bars indicate the ratio of RVFV IgG-seropositive camels to total camels in that age and sex category. (**B**) Human RVFV IgG seroprevalence stratified by age (<18 years old, ≥18 years old) and sex. Numbers above bars indicate the ratio of RVFV IgG-seropositive individuals to total individuals in that age and sex category. Two adult males were also identified as IgG-equivocal and are not included in the bar plot.

Of the 493 camel sera specimens, 9 (all IgG negative) were missing either age or sex information; therefore, 484 specimens were used for stratifying seroprevalence by age and sex. Female camel seroprevalence was 18.2% (68/372) and all RVFV-seropositive female camels were adults (Figure 3A). The highest rates of seropositive female camels occurred within the ages of 8, 7 and 4 year-old female adults and most seropositive female camels spanned a timeframe from 2005 to 2015 (Supplementary Figure S2A–D). Only one male calf (5.8%, 1/17) and six adult male camels (9.2%, 6/65) were RVFV-seropositive (Figure 3A, Supplementary Figure S2D). Overall, the dataset had fewer male camels (*n* = 82) than female camels (*n* = 402), because male camels are typically removed from the herd prior to reaching adulthood. All camel serum samples were also assessed for the presence of RVFV RNA by qRT-PCR and all were negative.

Human camel owners were also evaluated for the presence of RVFV-specific IgG antibodies (Figure 3B). A total of 197 human sera specimens were assessed for the presence of anti-RVFV IgG antibodies; 7.6% (15/197) were positive, and two adult males were equivocal (Figure 3B). RVFV seroprevalence was highest in males over 18 years (13.9%, 13/93) and two female owners above and below age 18 (4%, 1/25 ≤18 yo and 4%, 1/25 >18 yo) were RVFV-seropositive (Figure 3B).

### 3.3. Analysis of Factors Associated with RVFV IgG Seroprevalence

A univariate analysis was conducted to evaluate for the presence of factors associated with RVFV IgG seroprevalence among camels and human owners. Herd type (main herd vs. mother/home herd) was not significantly associated with RVF IgG seropositivity, but female camels were 2.26 times more likely to be seropositive (*p* = 0.046) (Table 1). Herd sizes above 50 animals were 2.54–3.37 times more likely to be RVF IgG-seropositive (*p* = 0.006 and *p* < 0.005) and there was no significant difference between herd sizes of 50–100 or greater than 100 (*p* = 0.36). There was also a statistically significant difference between a herd location with low RVF IgG positivity (location 4) and locations with high RVFV IgG seropositivity [locations 2 (29.49 times more likely), 6 (12.32×) and 7 (3.10×)] (Table 1). For the camel human owners, individuals engaged in farming-related activities were 6.16 times more likely to be seropositive (*p* = 0.03) and those >18 years old were 5.52 times more likely to be seropositive (*p* = 0.01). In contrast, human owner sex (*p* = 0.36) and reported camel contact (*p* = 0.70) were not significantly associated with human RVFV IgG seropositivity (Table 2).

### 3.4. Spatial Distribution of RVFV Seropositivity in Camels and Humans

**Figure 4:**
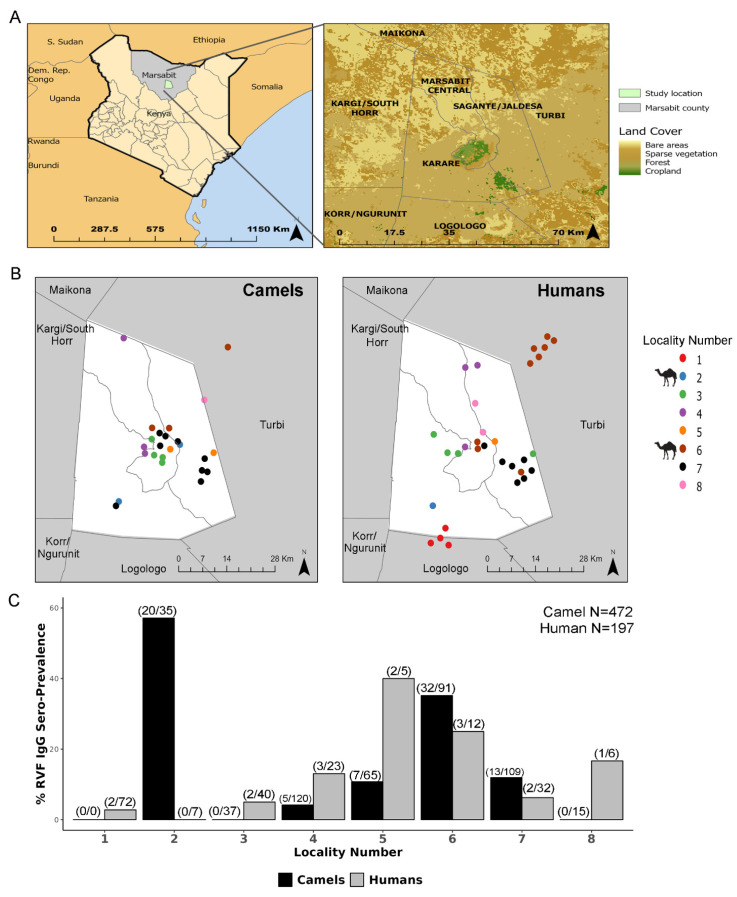
Geospatial distribution of RVF IgG seroprevalence in camels and humans. (**A**) Map of Marsabit county (grey) and the study site (green) (left panel). Right panel illustrates land cover in the study area. (**B**) Locations of camel and huma specimen localities in Marsabit county, Kenya. Camel icons indicate IgG seroprevalence greater than 20% in the legend. (**C**) Human and camel RVF IgG seroprevalence versus locality number. Numbers in parentheses represent total number of RV IgG-seropositive individuals versus total number of individuals in each locality number.

Sampling site locations of camels and human owners occurred in regions around Marsabit county, Saku sub-county, Kenya, which contains both sparse and forest/cropland regions (Figure 4A). Camels and humans were enrolled at different time points throughout the original MERS-CoV2 study; therefore, closeness of geocodes at enrollment, herds that grazed together, and similarity in household member details were used to link camel and humans to a locality number (Figure 4B). Camels from location 2 (57.14%, 20/35) and location 6 (35.16%, 32/91) had greater than 20% RVFV IgG seropositivity (Figure 4C). The highest human seropositivity was observed at location 5 (40%, 2/5) and location 6 (25%, 3/12) (Figure 4C).

## 4. Discussion

As One Health and global health security become increasing priorities, the necessity for high-quality diagnostics to detect previous or current infections of zoonotic arboviral disease in livestock species, such as camels, is increasing. Here, a previously established human RVFV IgG-specific ELISA [19,20] was modified to detect RVFV-specific camel IgG. The RVFV camel- and human-specific assays detected 15.6% of camels and 7.6% of camel human owners from Marsabit county Kenya as RVFV-seropositive. Large herd size, female camels, and location were significantly associated with camel RVFV IgG seropositivity; for camel human owners, farming-related activities and an age greater than 18 years old were associated with RVFV IgG seropositivity. The majority of seropositive camels and humans were associated with a small number of location groups (6, 2, and 5), which are located in cropland and forest areas in the central Karere ward of the Marsabit district.

The rationale for developing a novel RVFV-specific ELISA, despite the presence of commercial RVFV-specific ELISAs, was to develop a serological tool with distinct characteristics beyond what is readily available. Both mass-produced RVFV-specific ELISAs (IDVet and BDSL) rely on recombinant RVFV nucleocapsid for antibody screening [27,28,29,30,31]. In contrast, the ELISA developed here uses RVFV antigens produced during viral infection of Vero E6 cells, thus allowing for the detection of both structural (NP, Gn, Gc, L) and non-structural proteins (NSs and NSm). Additionally, the ELISA presented here includes a normalization step to subtract out the contribution of non-specific antigens detected due to antigen preparation methods (for example, Vero E6 antigens). This additional step is not included in the commercially available ELISAs, thus a positive reaction on these assays could be due to non-specific antigen reactivity. Finally, competitive ELISAs rely on the presence of a primary antibody to block the binding of an RVFV-specific antibody and thus may miss primary antibodies that bind to different epitopes and still permit the binding of the RVFV-specific antibodies—therefore causing a false-negative reaction. Altogether, the RVFV-specific indirect ELISA presented here introduces an expanded detection capacity beyond what is currently commercially available; future studies should explore the specificity differences between ELISAs manufactured with recombinant, single viral antigens versus all available viral antigens.

The camel RVFV IgG seroprevalence identified here and associated factors suggest that RVFV infection among camels in Marsabit, Kenya, primarily occurs in large herds near cropland/forested areas and that exposure increases with age (since females were predominantly infected and males are culled from the herd at a young age). The highest rates of seropositive animals spanned a timeframe from 2005 to 2015; this timeframe coincides with a large RVFV outbreak in Kenya in 2006–2007 [32] and a period with heavy El-Niño-associated rainfall in 2015 (that was not associated with increased RVFV seropositivity in southern Kenya) [33]. In March–May 2018, heavy rainfall in northeastern Kenya resulted in increased RVFV monitoring, which identified mass abortions and mortality of young sheep, camels, and goats in neighboring Wajir county [34]. Overall 30 laboratory-confirmed RVFV-positive human cases were identified in May–June 2018—21 from neighboring Wajir and Siaya counties and 8 from Marsabit [34]. The first human case is believed to have been an individual from Wajir county that consumed meat from a sick camel [34]. The camel specimens evaluated here were collected between 31 July and 10 August 2018—the low rates of seropositive 0–3 year-old camels suggests that a naïve camel population was available in Marsabit county to sustain the RVFV infections that started in neighboring Wajir county in March–May of 2018.

An association between age and camel RVFV seropositivity and rainfall amounts was also observed among camels from Khartoum State, Sudan [35]. Interestingly, in Khartoum State, Sudan, there was not a significant association between human handler seropositivity and camel contact, only a significant association between human seropositivity and farming-related activities, which suggests that human handlers were not significantly infected with RVFV due to camel-related contact. Farmers participating in the Sudan study reported rearing camels and at least one other livestock, such as goats, cattle/sheep or donkeys [8], suggesting other mechanisms for farm-related RVFV infections and exposures beyond camel-related interactions (such as an increase in infected mosquitoes after prolonged rain and the presence of other susceptible species, such as cattle, sheep and goats). In Marsabit county, a similar significant association between farming-related activities (but not camel contact) and RVFV human owner seropositivity was observed, demonstrating that other farm-related exposures, in addition to camel handling, can lead to human RVFV infections.

In Kenya, RVFV camel infection was identified as early as 1962 following heavy rainfall and abortions reported in cattle and camels; specifically, 46% (14/30) of camels from Marsabit county in 1962 were RVFV-seropositive [36]. Since that time RVFV camel seroprevalence has varied between 22% in southern Kenya in 1985 [37], 20.9% in central Kenya in 2017 [32], 13.5–14.2% in Samburu and Isiolo counties south of Marsabit county in 2020, 2021, and 2023 [38,39,40], and 47% in April 2025 [41]. Neighboring countries report 28% and 43% camel RVFV seroprevalence in Tanzania and Ethiopia, respectively [42,43]. In October 2010 unprecedented rainfall in Mauritania lead to an increase in the mosquito population and the identification of RVFV-positive human and camel cases [44]. It is not currently clear whether RVFV causes clinical disease in camels [45]; the 2010 Mauritania outbreak reported evidence of clinical disease [44], yet a previous study observed no evidence of disease for RVFV-positive camels [37]. Altogether, the serological data and data from previous RVFV outbreaks demonstrate that RVFV (or an antigenically similar Phlebovirus) infects camels across eastern Africa. No previous studies have explored the association between RVFV-seropositive camels and the risks to the camel handlers.

As farmers transition away from watering-intensive animals, such as cattle and goats, to drought-resistant livestock, such as camels, disease surveillance efforts need to transition, too. In 2014, interviews among farmers from the Isiolo district reported that 71.5% preferred raising camels because they were well-suited for the region, can stay for many days without water and food, and also are usable for transport [46]. Camels were also reported as the first-choice livestock species among 98% of Somali pastoralists, where camels serve as the milk, meat and draft power for local pastoralists [47]. In 2024, an RVFV outbreak was reported in Marsabit and Wajir counties in Kenya that infected both individuals, as well as causing camel and other animal deaths [48]. Media reports suggested that the index case consumed and shared meat from a sick camel prior to developing RVFV illness [49].

In this study, a camel-specific RVFV IgG ELISA demonstrated that dromedary camels in Marsabit, Kenya, in 2018 were serologically positive for RVFV six years before an RVFV outbreak occurred in humans, camels and other animals in the Marsabit region (in 2024). As local farmers transition from cattle-based farming to camel-based farming, the lack of unestablished health and safety guidelines for camel farming leaves Kenya vulnerable to camel-borne zoonotic diseases; for example, the Kenya Meat Commission does not currently have guidelines for camel meat abattoirs. With the development of novel RVFV human and livestock vaccines [50,51], the possibility of limiting regional and district viral spread, while also supporting the local camel economy, exists. Here, this study demonstrates that camels can serve as an intermediate species capable of amplifying and spreading the vector-borne disease.

## Figures and Tables

**Table 1:** Univariate analysis of factors associated with camel RVF IgG seropositivity. Total specimen count used for the analyses is in parentheses—of the 493 camel sera specimens, several were missing variable information.

Variable	Total, *n*	Seropositive, *n* (%)	OR (95%CI)	Fisher’s *p*-Value	χ^2^ *p*-Value
Herd Type (*n* = 470)					
Main Herd	285	43 (15.1%)	0.78 (0.46–1.33)	0.373	0.346
Mother/Home Herd	185	34 (18.4%)			
Herd Size (*n* = 473)					
Less than 50	230	21 (9.1%)	Reference		0.0001
50–100	113	23 (20.4%)	2.54 (1.27–5.09)	0.006	
Greater than 100	130	33 (25.4%)	3.37 (1.79–6.48)	<0.005	
Sex (*n* = 467)					
Female	387	69 (17.8%)	2.26 (0.98–6.07)	0.046	0.045
Male	80	7 (8.8%)			
Age (*n* = 470)					
Adult (12 mos+)	446	75 (16.8%)	4.64 (0.73–193.9)	0.151	0.101
Calf (0–12 mos)	24	1 (4.2%)			
Herd Location (*n* = 472)					
2	35	20 (57.1%)	29.48 (9.10–116.15)	<0.005	
3	37	0 (0%)	0 (0–3.55)	0.592	
4	120	5 (4.2%)	Reference		
5	65	7 (10.8%)	2.76 (0.72–11.53)	0.110	
6	91	32 (35.2%)	12.32 (4.45–42.68)	<0.005	
7	109	13 (11.9%)	3.10 (0.99–11.51)	0.047	
8	15	0 (0%)	0 (0–9.15)	1.000	

**Table 2:** Univariate analysis of factors associated with human RVF IgG seropositivity (*n* = 195).

Variable	Total *, *n*	Seropositive, *n* (%)	OR (95%CI)	Fisher’s *p*-Value
Occupation (*n* = 195)				
Farming-Related	113	13 (11.5%)	5.16 (1.11–48.46)	0.03
Other	82	2 (2.4%)		
Sex (*n* = 195)				
Female	50	2 (4.0%)	0.42 (0.04–1.98)	0.36
Male	145	13 (9.0%)		
Age (*n* = 195)				
>18 yrs	110	13 (11.8%)	5.52 (1.20–51.80)	0.01
<=18 yrs	85	2 (2.3%)		
Camel Contact (*n* = 195)				
No	26	1 (3.8%)	0.44 (0.01–3.18)	0.70
Yes	169	14 (8.3%)		

* Two RVFV-Equivocal (i.e., nearly positive) individuals were not included in the analysis.

## Data Availability

An anonymized, de-identified version of the dataset can be made available on reasonable request to the corresponding author.

## Supplementary Materials

The following supporting information can be downloaded at: https://www.mdpi.com/article/10.3390/v18040445/s1. Figure S1: Optimization of RVFV-specific camel IgG ELISA; Figure S2: Distribution of RVFV-seropositive and -seronegative camel ages.
