# Supplementary material for: A Newly Established ELISA for the Surveillance of Rift Valley Fever in Dromedary Camels and Their Owners, Kenya 2018
Source: Viruses. 2026 Apr 8;18(4):445. doi: 10.3390/v18040445 (PMC13119942; doi:10.3390/v18040445)
Supplement: Supplementary file 1 [file viruses-18-00445-s001.zip › Supplemental Figure Legends.pdf]

Supplemental Figure Legends

Supplemental Figure 1: Optimization of RVFV-specific camel IgG ELISA. **A)** Distribution of adjusted optical density (OD) values for all dilutions from initial protein A/G-HRP screening using a subset (n=93) of available camel sera specimens. Dashed red line represents signal intensity threshold set at three standard deviations above background (orange points) and negative control camel (blue points) adjusted OD values. **B)** Distribution of adjusted optical density OD values for summarized dilutions ("Sum OD") from initial protein A/G-HRP screening using a subset (n=93) of available camel sera specimens. Dashed red line represents signal intensity threshold set in as in A. **C)** Distribution of adjusted optical density (OD) values for summarized dilutions from all available camel sera specimens, detected with anti-Llama HRP. Dashed red line represents signal intensity threshold set as in A.

Supplemental Figure 2: Distribution of RVFV seropositive and seronegative camel ages. Histogram bars encompass left-biased values such that the left edges of bins are included in the bin. **A)** Distribution of ages for all tested camels. **B)** Distribution of ages for all RVFV ELISA-positive camels. **C)** Distribution of ages for all RVFV ELISA-positive female camels. **D)** Distribution of RVFV ELISA-positive male camels.
