# Supplementary figures and images for: A Newly Established ELISA for the Surveillance of Rift Valley Fever in Dromedary Camels and Their Owners, Kenya 2018
Source: Viruses. 2026 Apr 8;18(4):445. doi: 10.3390/v18040445 (PMC13119942; doi:10.3390/v18040445)

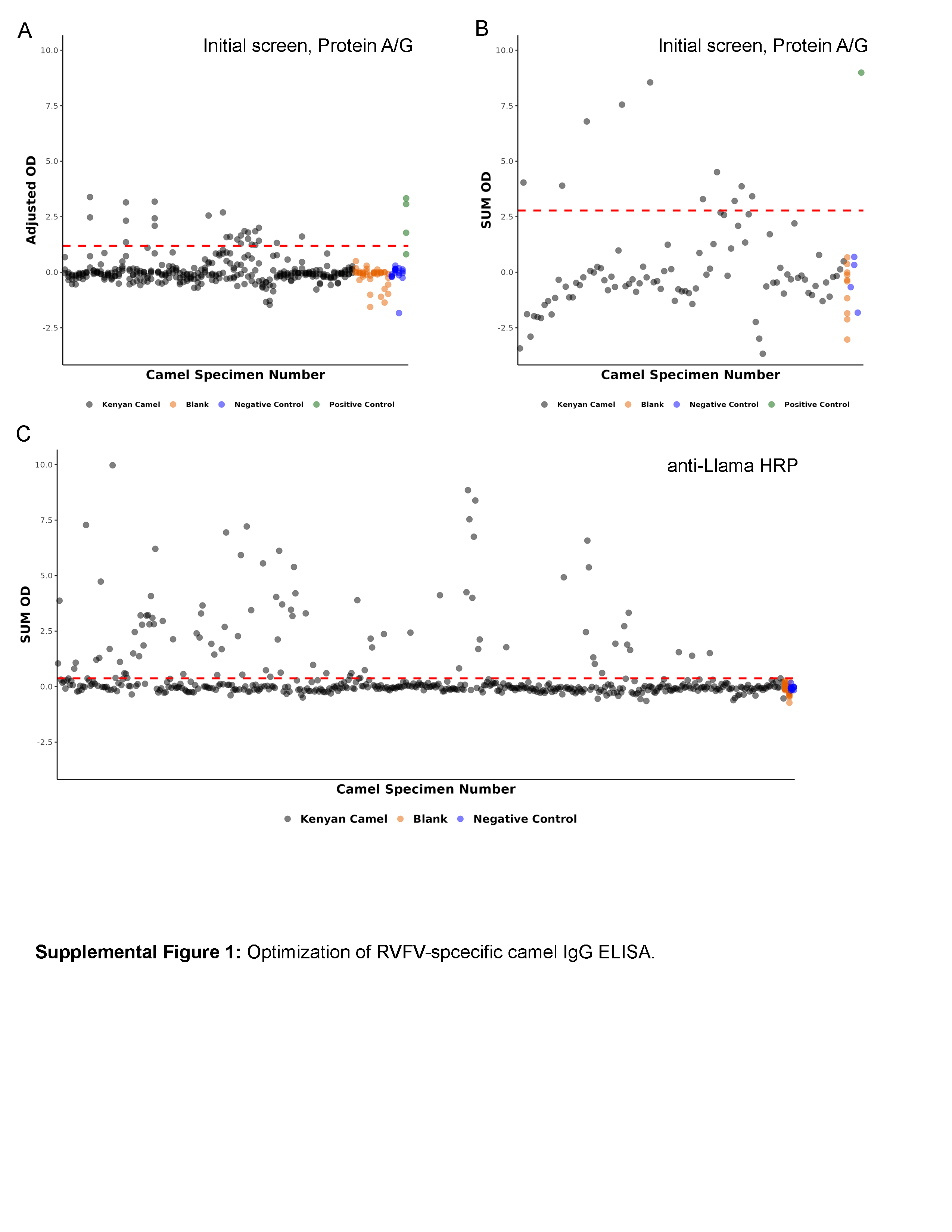

Supplement: Supplementary file 1 [file viruses-18-00445-s001.zip › Supp_Figure1_V1_flat.tif]

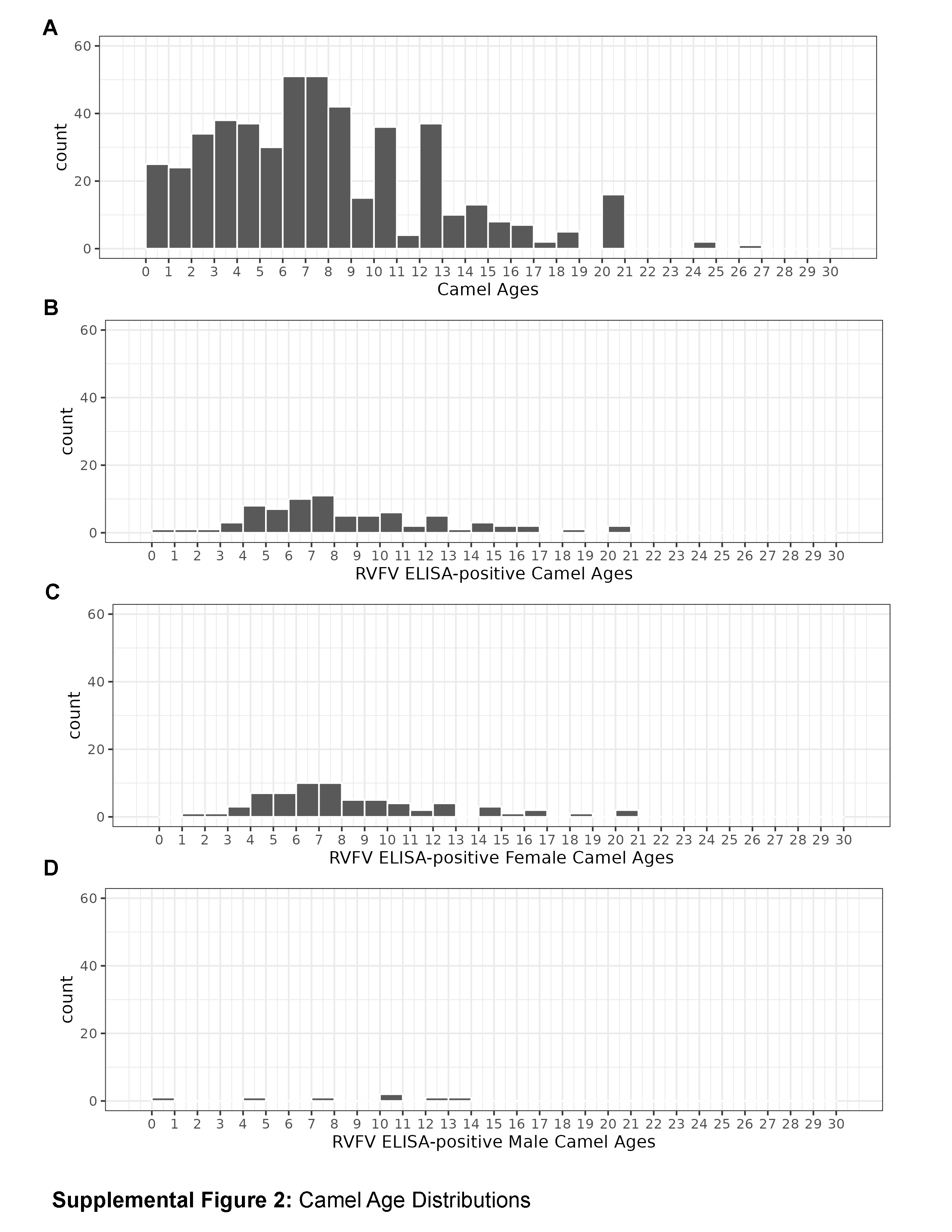

Supplement: Supplementary file 1 [file viruses-18-00445-s001.zip › Supp_Figure2_V1_flat.tif]
